# Structural Revision of Wentiquinone C and Related Congeners from Anthraquinones to Xanthones Using Chemical Derivatization and NMR Analysis

**DOI:** 10.3390/md17010008

**Published:** 2018-12-24

**Authors:** Xin Li, Xiao-Ming Li, Bin-Gui Wang

**Affiliations:** 1 Key Laboratory of Experimental Marine Biology, Institute of Oceanology, Chinese Academy of Sciences, Laboratory of Marine Biology and Biotechnology, Pilot National Laboratory for Marine Science and Technology (Qingdao), Nanhai Road 7, Qingdao 266071, China; lixin871014@163.com (X.L.); lixmqdio@126.com (X.-M.L.); 2 Center for Ocean Mega-Science, Chinese Academy of Sciences, Nanhai Road 7, Qingdao 266071, China

**Keywords:** Structure revision, Wentiquinone C, Methylation method, Xanthones, Anthraquinones, *Aspergillus wentii*

## Abstract

Wentiquinone C, which was previously isolated from the marine brown alga-derived endophytic fungus *Aspergillus wentii* EN-48, was found to be a potent antioxidant against α,α-diphenyl-picrylhydrazyl (DPPH) radical. The structure of wentiquinone C was originally assigned as an anthraquinone derivative (1,10-dihydroxy-3-(hydroxymethyl)-8-methoxydibenzo [*b*,*e*]oxepine-6,11-dione, **1**) by 1D and 2D NMR experiments. However, the minor differences of the chemical shifts between xanthones and anthraquinones were queried, leading to the structure of **1** to be revised as a xanthone analog (8-hydroxy-6-(hydroxymethyl)-3-methoxy-9-oxo-9*H*-xanthene-1-carboxylic acid, **2**) on the basis of a methylation and subsequent NMR measurements, and was confirmed by X-ray crystallographic analysis. The method established in this paper could be applied to the structural re-examination or revision for some of the reported *seco*-anthraquinone derivatives.

## 1. Introduction

**Figure 1:**
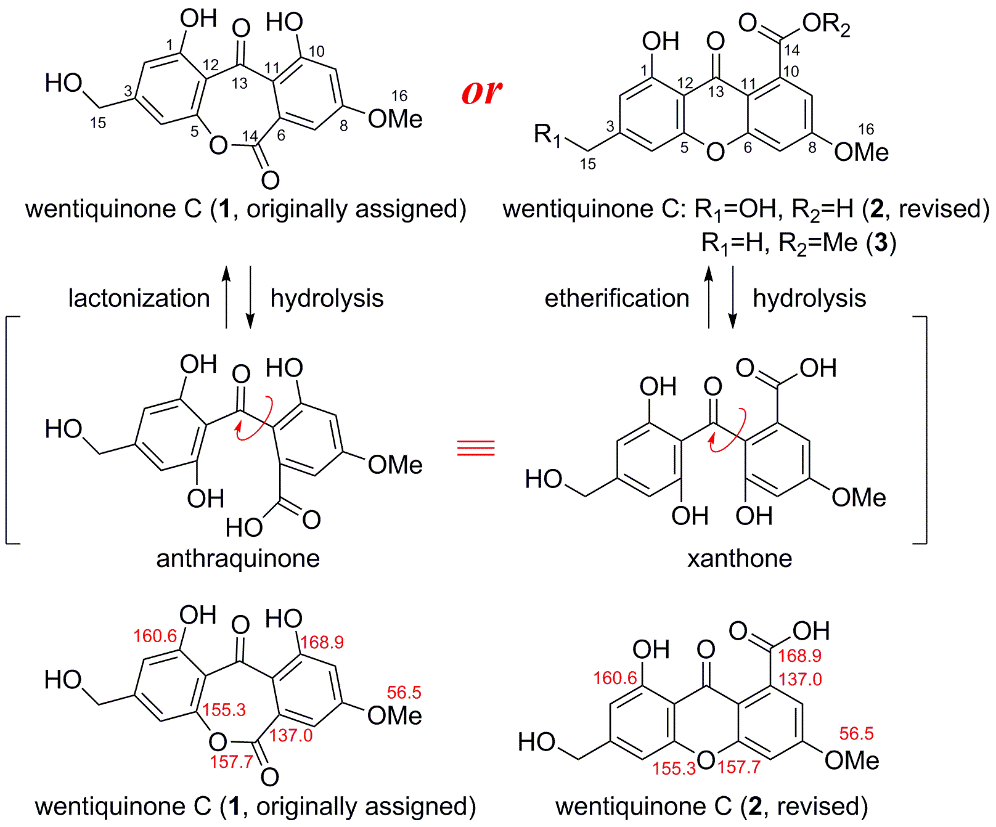
Two possible structures for wentiquinone C (**1** and **2**) and their characteristic ^13^C NMR data assignments (in DMSO-*d*_6_) as well as the reference compound **3**.

In the course of our chemical investigation of marine-derived fungi for bioactive metabolites, we have isolated and assigned the structure of wentiquinone C as an anthraquinone derivative from the marine brown algal-derived endophytic fungus *Aspergillus wentii* EN-48, and the compound showed potent antioxidant activity against α,α-diphenyl-picrylhydrazyl (DPPH) radicals [1]. Based on the NMR analysis, its structure was originally assigned as 1,10-dihydroxy-3-(hydroxymethyl)-8- methoxydibenzo[*b*,*e*]oxepine-6,11-dione (anthraquinone derivative, **1**), which contained a seven-membered lactone ring in the structure (Figure 1). In our continuing investigation for more bioactive secondary metabolites from the same fungal strain, compound **3** was characterized, and its ^1^H and ^13^C NMR spectral data were very similar to those of wentiquinone C, indicating that **3** might be an analog of wentiquinone C. However, the structure of **3** was finally assigned to be calyxanthone, a xanthone derivative that was firstly discovered in 1983, according to its NMR data [2]. Compounds **1** and **3** have very similar NMR data but belonged to anthraquinone and xanthone derivatives, respectively, and this phenomenon promoted us to query if there is other possible structure assigned for wentiquinone C (**1**) as a xanthone (**2**). Meanwhile, Yenesew and co-workers proposed the revision for a group of *seco*-anthraquinones to xanthones [3], and suggested that the structure of wentiquinone C should also be revised. Nevertheless, it was challenging to distinguish **1** and **2** based only on the MS and NMR data because they possessed the same carbon connectivity, similar NMR chemical shifts, and identical HMBC correlations. To clear up this confusion, a methylation and subsequent NMR analysis were employed successfully, and leading to unambiguously revision of the structure of wentiquinone C from an anthraquinone derivative (**1**) to a xanthone analogue (**2**) (Figure 1). This paper details the structure revision of wentiquinone C and other related natural products.

## 2. Results and Discussion

The different ways of cyclization led to two possible assignments for the structure of wentiquinone C: one contained a seven-membered lactone (anthraquinone derivative, **1**), and another possessed a six-membered ether ring instead (xanthone analog, **2**), which having free carboxyl group (Figure 1). The ^13^C NMR chemical shifts were seemingly reasonable for both assignments, except that in the case of **1**, the resonance assigned for the oxygenated quaternary carbon (C-10) at δ_C_ 168.9 was more downfielded than its usual value (around δ_C_ 162.0) [4] (Figure 1). The minor distinction implicated an incorrect assignment might be made during the structure elucidation of wentiquinone C [1].

**Figure 2:**
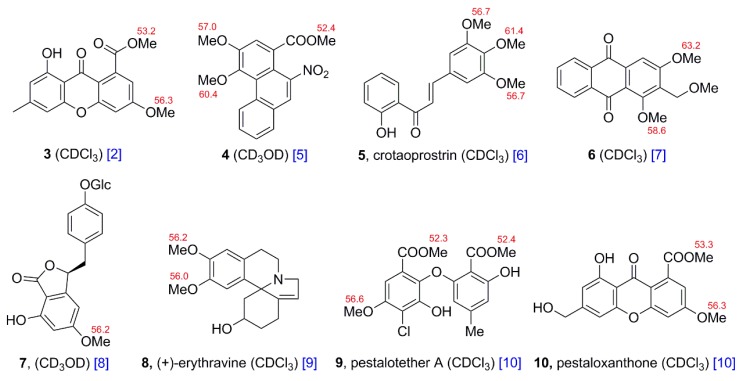
^13^C NMR chemical shifts for methoxy groups of compound **3** and some previously reported natural products with aromatic methoxy groups and/or methyl esters.

**Scheme 1:**
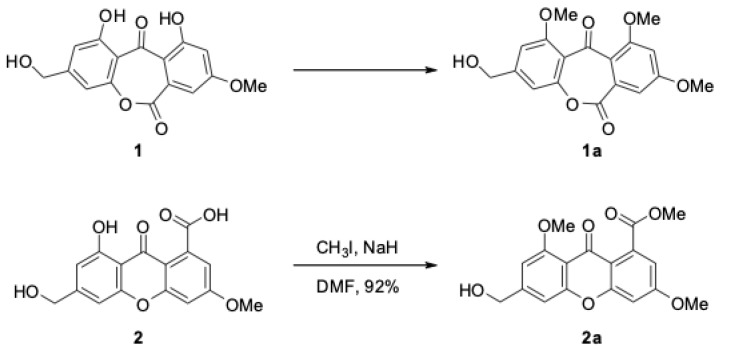
Synthesis of compound **2a**.

Herein, a methylation method, which applied to demonstrate the authentic structure of wentiquinone C, was established inspired by the struactually related analogue (**3**) (Figure 2). Two crucial methoxy groups in **3** were found to have different ^13^C NMR shifts, indicating that they are in different chemical environment. One of them is an aromatic methoxy group (δ_C_ 56.3), and another is a methyl ester (δ_C_ 53.2). A literature survey indicated the interesting distinction for the chemical shifts of aromatic methoxy and methyl ester groups [5,6,7,8,9,10] (Figure 2), which supported the undoubted difference in chemical shifts between the two types of methoxy groups. That is, in the ^13^C NMR spectrum the chemical shifts for aromatic methoxy groups are always bigger than δ_C_ 55.0, while those for methyl esters are usually smaller than δ_C_ 55.0. Thus, unlike wentiquinone C, the structure of **3** was unambiguous because of the existence of the methyl ester (δ_C_ 53.2), which revealed that the carboxyl had not participate in the cyclization. Similarly, it would be achievable to determine the structure of wentiquinone C by analyzing the shifts of methoxy groups after methylation for carboxyl or aromatic hydroxyl groups, and then the assignment of the intramolecular ring could be confirmed accordingly: for the case of structure 1, there should be three aromatic methoxy groups resonating at δ_C_ > 55 after methylation, while with respect to structure **2**, one methyl ester resonating at δ_C_ < 55 and two aromatic methoxy group resonating at δ_C_ > 55 should be observed (Scheme 1).

**Figure 3:**
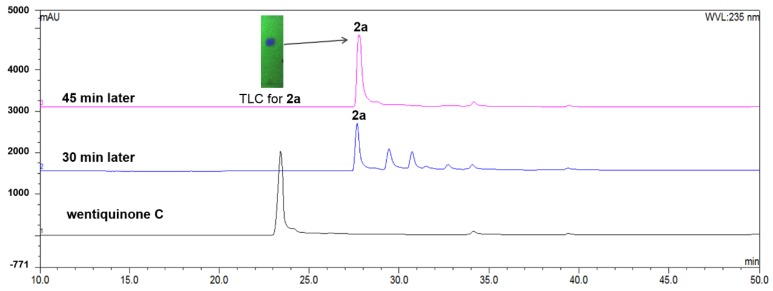
HPLC profiles for wentiquinone C and the reaction products at 30 and 45 min. Gradient: 0–5 min, 10% methanol in water; 6–35 min, gradient from 10% methanol in water to 100% methanol; 35–45 min, 100% methanol; 45–60 min, gradient from 100% methanol to 10% methanol in water. TLC developing solvent: CH_2_Cl_2_:MeOH = 10:1.

**Figure 4:**
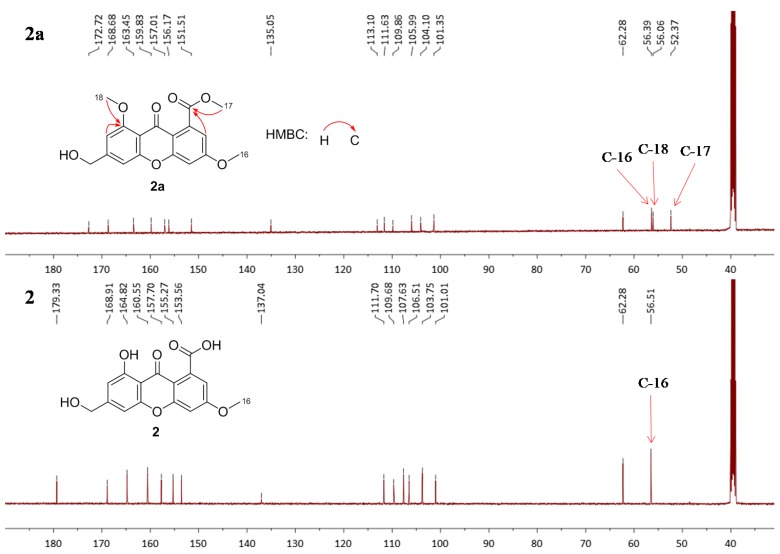
Comparison for ^13^C NMR spectra between compounds **2a** and **2** in DMSO-*d*_6_.

Methylation of wentiquinone C was achieved by treatment of the compound with CH_3_I in anhydrous DMF with the presence of NaH for 45 min [11]. The major product **2a** was obtained as yellowish amorphous powder in 92% yield (Figure 3). Its molecular formula was determined to be C_18_H_16_O_7_ by HRESIMS (*m/z* 345.0964 [M + H]^+^, calcd for C_18_H_17_O_7_^+^, 345.0969), with 28 unit (C_2_H_4_) more than that of wentiquinone C. The differences in the 1D NMR data between **2a** and wentiquinone C were that signals for two more methoxy groups at δ_C/H_ 52.4/3.85 (C/H-17) and 56.1/3.88 (C/H-18) were detected in **2a** (Figure 4), while the exchangeable proton of a phenolic hydroxyl group (δ_H_ 12.41) in wentiquinone C disappeared in the ^1^H NMR spectrum of 2a. As mentioned earlier, C-17 was observed at δ_C_ 52.4 and could be confirmed as a methyl ester, while C-18 was detected at δ_C_ 56.1 and should be an aromatic methoxy group (Figure 4). Therefore, it could be deduced that there is a carboxyl group in wentiquinone C, whose structure should be related to xanthone (**2**) rather than anthraquinone (**1**). This deduction was verified by the key HMBC correlations (Figure 4) from H-17 to C-14 and from H-18 to C-1.

**Figure 5:**
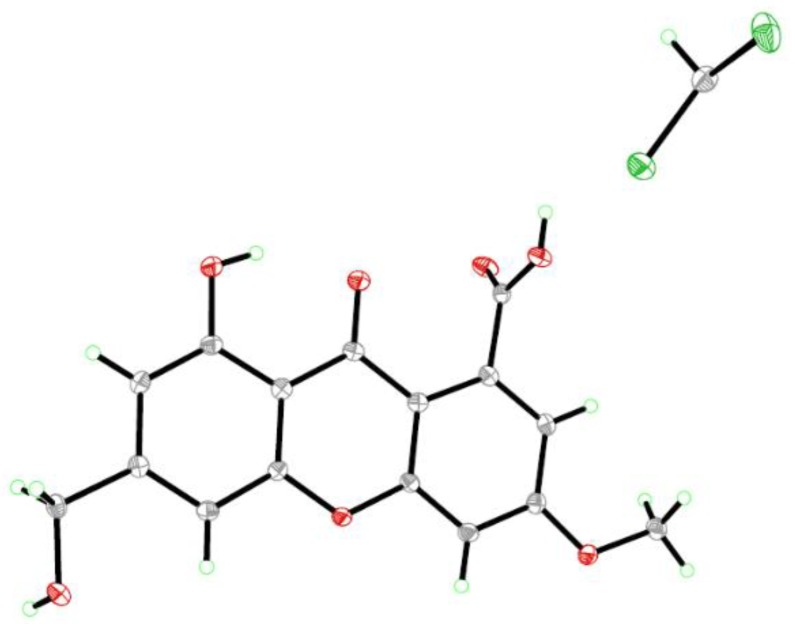
X-ray crystallographic structure of wentiquinone C (Note: The inclusive of CHCl_3_ was detected in the X-ray structure of wentiquinone C).

Slow evaporation of the solvents (MeOH:CHCl_3_ = 1:1) by keeping the sample of wentiquinone C in a refrigerator for one month yielded quality crystals suitable for X-ray crystallographic analysis, which unambiguously confirmed its structure (**2**) as shown in Figure 5. Thus, the structure of wentiquinone C should be revised to 2.

**Figure 6:**
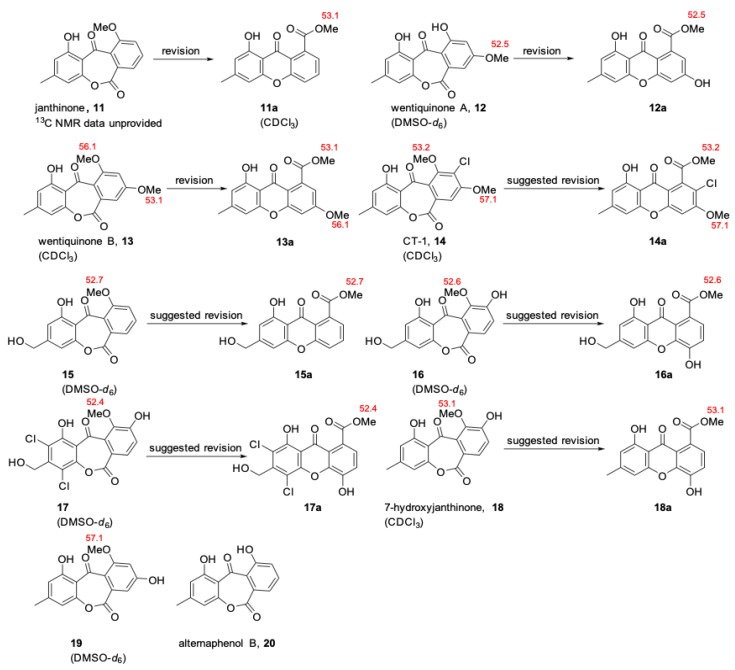
Assigned structures of *seco*-anthraquinones requiring reassessment as xanthones.

The results from a literature survey revealed that, besides wentiquinone C, the structures of several other noteworthy molecules of *seco*-anthraquinone class (Figure 6) need to be revisited, and the method established in this paper could be applied to the structural re-examining or revision for these compounds. For instance, janthinone (**11**) was originally reported as a *seco*-anthraquinones in 2005 [12], the structure of which has been revised to a xanthone by another group on the basis of X-ray crystallographic analysis [13]. The structures of some other natural products, such as wentiquinones A and B (**12** and **13**), CT-1 (**14**), and compounds **15**−**18**, which were initially assigned the structures as *seco*-anthraquinones [14,15,16,17,18], are likely incorrectly assigned and their structures should be reassessed. Compounds **19** and **20** [19,20], which were considered being *seco*-anthraquinones, are also required to be re-checked carefully.

## 3. Materials and Methods 

### 3.1. General Experimental Procedures

Melting points were determined with an SGW X-4 micromeltingpoint apparatus (Shanghai Shenguang Instrument Co. Ltd, Shanghai, China). UV spectra were measured on a PuXi TU-1810 UV-visible spectrophotometer (Shanghai Lengguang Technology Co. Ltd., Shanghai, China). 1D and 2D NMR spectra were obtained at 500 and 125 MHz for ^1^H and ^13^C, respectively, on a Bruker Avance 500 MHz spectrometer (Bruker Biospin Group, Karlsruhe, Germany) with tetramethyl silane (TMS) as an internal standard. Mass spectra were generated on a VG Autospec 3000 (VG Instruments, London, UK) or an API QSTAR Pulsar 1 mass spectrometer (Applied Biosystems, Foster, Waltham, MA, USA). Analytical and semipreparative HPLC were performed using a Dionex HPLC system equipped with a P680 pump, an ASI-100 automated sample injector, and a UVD340U multiple wavelength detector controlled by Chromeleon software (version 6.80) (Dionex, Sunnyvale, CA, USA). Commercially available Si gel (200–300 mesh, Qingdao Haiyang Chemical Co., Qingdao, China), Lobar LiChroprep RP-18 (40–63 μm, Merck, Darmstadt, Germany), and Sephadex LH-20 (Pharmacia, Pittsburgh, PA, USA) were used for open column chromatography. All solvents were distilled prior to use.

### 3.2. Methylation of Wentiquinone C

A suspension of NaH (1.0 mg) in 1.0 mL of anhydrous DMF was treated with a solution of wentiquinone C (3.0 mg) in 3.0 mL of DMF at 0 °C. The reaction mixture was stirred at 0 °C for 30 min, and then CH_3_I (30 μL) was added. After 45 min, a saturated solution of NH_4_Cl (10.0 mL) was added to quench the reaction. The product was extracted with EtOAc (4 × 10 mL) and purified by HPLC to give 2a (3.0 mg, 92%).

### 3.3. Wentiquinone C (***2***)

Yellow crystals; m.p. 287–289 °C; UV (MeOH) *λ*_max_ (log *ε*) 236 (4.61), 247 (4.55), 305 (4.28), 354 (3.93) nm; ^1^H NMR(DMSO-*d*_6_, 500 MHz) δ 6.75 (1H, brs, H-2), 6.95 (1H, brs, H-4), 7.16 (1H, d, *J* = 2.0 Hz, H-7), 6.93 (1H, d, *J* = 2.0 Hz, H-9), 4.59 (2H, s, H-15), 3.94 (3H, s, H-16), 12.41 (1H, s, 1-OH); ^13^C NMR (DMSO-*d*_6_, 125 MHz) δ 160.6 (C-1, C), 107.6 (C-2, CH), 153.6 (C-3, C), 103.8 (C-4, CH), 155.3 (C-5, C), 157.7 (C-6, C), 100.8 (C-7, CH), 164.8 (C-8, C), 111.7 (C-9, CH), 137.0 (C-10, C), 109.7 (C-11, C), 106.5 (C-12, C), 179.4 (C-13, C), 168.9 (C-14, C), 62.3 (C-15, CH_2_), 56.5 (C-16, CH_3_). HRESIMS *m/z* 317.0639 ([M + H]^+^) (calculated for C_16_H_13_O_7_^+^,317.0656).

### 3.4. Compound ***2a***

Yellow amorphous powder; UV (MeOH) *λ*_max_ (log *ε*) 235 (4.53), 248 (4.45), 303 (4.15), 350 (3.86) nm; ^1^H NMR (DMSO-*d*_6_, 500 MHz) δ 6.94 (1H, s, H-2), 7.06 (1H, s, H-4), 7.17 (1H, s, H-7), 6.94 (1H, s, H-9), 4.63 (2H, d, *J* = 5.6 Hz, H-15), 3.92 (3H, s, H-16), 3.85 (3H, s, H-17), 3.88 (3H, s, H-18), 5.54 (1H, brs, 15-OH); ^13^C NMR(DMSO-*d*_6_, 125 MHz) δ 159.8 (C-1, C), 104.1 (C-2, CH), 151.5 (C-3, C), 106.0 (C-4, CH), 157.0 (C-5, C), 156.2 (C-6, C), 101.4 (C-7, CH), 163.4 (C-8, C), 111.6 (C-9, CH), 135.0 (C-10, C), 113.1 (C-11, C), 109.9 (C-12, C), 172.7 (C-13, C), 168.7 (C-14, C), 62.3 (C-15, CH_2_), 56.4 (C-16, CH_3_), 52.4 (C-17, CH_3_), 56.1 (C-18, CH_3_). HRESIMS *m/z* 345.0964 ([M + H]^+^) (calculated for C_18_H_17_O_7_^+^, 345.0969).

### 3.5. X-ray Crystallographic Analysis of Wentiquinone C (***2***)

All crystallographic data were collected on an Agilent Xcalibur Eos Gemini CCD plate diffractometer equipped with a graphite-monochromatic Cu K*α* radiation (λ = 1.54178 Å) at 293(2) K. The data were corrected for absorption by using the program SADABS [21]. The structure was solved by direct methods with the SHELXTL software package [22]. All nonhydrogen atoms were refined anisotropically. The H atoms were located by geometrical calculations, and their positions and thermal parameters were fixed during the structure refinement. The structure was refined by full-matrix least-squares techniques [23].

### 3.6. Crystal Data for Wentiquinone C (***2***)

C_32_H_24_O_14_·CHCl_3_, FW = 751.88, monoclinic space group C2/c, unit cell dimensions *a* = 12.9467(7) Å, *b* = 10.9982(8) Å, *c* = 22.0459(15) Å, V = 3041.8(3) Å3, *α* = 90°, *β* = 104.302(2)°, *γ* = 90°, Z = 4, *d*_calcd_ = 1.642 mg/m3, crystal dimensions 0.42 mm × 0.40 mm × 0.25 mm, *μ* = 3.414 mm^−1^, *F*(000) = 1544. The 9808 measurements yielded 2675 independent reflections after equivalent data were averaged, and Lorentz and polarization corrections were applied. The final refinement gave *R*_1_ = 0.0431 and *w*R_2_ = 0.1156 [*I* > 2*σ*(*I*)]. Crystallographic data of wentiquinone C (**2**) have been deposited in the Cambridge Crystallographic Data Centre as CCDC 1406785. The data can be obtained free of charge via http://www.ccdc.cam.ac.uk/data_request/cif (or from the CCDC, 12 Union Road, Cambridge CB21EZ, UK; fax: +44-1223-336-033; e-mail: deposit@ccdc.cam.ac.uk).

## 4. Conclusions

In conclusion, the structure of wentiquinone C was revised to be 8-hydroxy-6-(hydroxymethyl)-3-methoxy-9-oxo-9H-xanthene-1-carboxylic acid (**2**) by a methylation method and confirmed by an X-ray diffraction study. The characteristic differences between aromatic methoxy group and methyl ester were discussed here based on the literatures data, and the methylation method has proven to be useful to rectify such a misassignment for structurally related compounds.

## References

[1] Li X., Li X.M., Xu G.M., Li C.S., Wang B.G. (2014). Antioxidant metabolites from marine alga-derived fungus *Aspergillus wentii* EN-48. Phytochem. Lett..

[2] Hamasaki T., Kimura Y. (1983). Isolation and structures of four new metabolites from *Aspergillus wentii*. Agric. Biol. Chem..

[3] Abdissa N., Heydenreich M., Midiwo J.O., Ndakala A., Majer Z., Neumann B., Stammler H., Sewald N., Yenesew A. (2014). A xanthone and a phenylanthraquinone from the roots of *Bulbine frutescens*, and the revision of six *seco*-anthraquinones into xanthones. Phytochem. Lett..

[4] Hein S.M., Gloer J.B., Koster B., Malloch D. (1998). Arugosin F: A new antifungal metabolite from the coprophilous fungus *Ascodesmis sphaerospora*. J. Nat. Prod..

[5] Mizuno M., Oka M., Iinuma M., Tanaka T. (1990). An aristolochic acid derivative of *Aristolochia liukiuensis*. J. Nat. Prod..

[6] Krohn K., Steingröver K., Srinivasa Rao M. (2002). Isolation and synthesis of chalcones with different degrees of saturation. Phytochemistry.

[7] Chiriboga X., Gilardoni G., Magnaghi I., Finzi P.V., Zanoni G., Vidari G. (2003). New anthracene derivatives from *Coussarea macrophylla*. J. Nat. Prod..

[8] Zidorn C., Grass S., Ellmerer E.P., Ongania K., Stuppner H. (2006). Stilbenoids from *Tragopogon orientalis*. Phytochemistry.

[9] Flausino O., Santos L.A., Verli H., Pereira A.M., Bolzani V.S., Nunes-de-Souza R.L. (2007). Anxiolytic effects of erythrinian alkaloids from *Erythrina mulungu*. J. Nat. Prod..

[10] Klaiklay S., Rukachaisirikul V., Tadpetch K., Sukpondma Y., Phongpaichit S., Buatong J., Sakayaroj J. (2012). Chlorinated chromone and diphenyl ether derivatives from the mangrove-derived fungus *Pestalotiopsis* sp. PSU-MA69. Tetrahedron.

[11] Fu P., Wang S.X., Hong K., Li X., Liu P.P., Wang Y., Zhu W.M. (2011). Cytotoxic bipyridines from the marine-derived actinomycete *Actinoalloteichus cyanogriseus* WH1-2216-6. J. Nat. Prod..

[12] Marinho A.M.R., Rodrigues-Filho E., Moitinho M.L.R., Santos L.S. (2005). Biologically active polyketides produced by *Penicillium janthinellum* isolated as an endophytic fungus from fruits of *Melia azedarach*. J. Braz. Chem. Soc..

[13] Shao C., Wang C., Wei M., Gu Y., Xia X., She Z., Lin Y. (2008). Structure elucidation of two new xanthone derivatives from the marine fungus *Penicillium* sp. (ZZF 32#) from the South China Sea. Magn. Reson. Chem..

[14] Sun H.F., Li X.M., Meng L.H., Cui C.M., Gao S.S., Li C.S., Wang B.G. (2013). Two new *seco*-anthraquinone derivatives from the Marine-derived endophytic fungus *Aspergillus wentii* EN-48. Helv. Chim. Acta.

[15] Fujimoto H., Inagaki M., Satoh Y., Yoshida E., Yamazaki M. (1996). Monoamine oxidase-inhibitory components from an ascomycete, *Coniochaeta tetraspora*. Chem. Pharm. Bull..

[16] Carvalho M.R., de Almeida Barbosa L.C., de Queiróz J.H., Howarth O.W. (2001). Novel lactones from *Aspergillus versicolor*. Tetrahedron Lett..

[17] Chunyu W.X., Zhao J.Y., Ding Z.G., Wang Y.X., Han X.L., Li M.G., Wen M.L. (2018). A new dichlorinated aromatic lactone from the tin mine tailings-derived fungus *Torula* sp. YIM DT 10072. Chem. Nat. Compd..

[18] Guo Z., Cheng F., Zou K., Wang J., She Z., Lin Y. (2009). Secondary metabolites from the mangrove endophytic fungus *Penicillium* sp. (SBE-8). Nat. Prod. Commun..

[19] Pan J.H., Deng J.J., Chen Y.G., Gao J.P., Lin Y.C., She Z.G., Gu Y.C. (2010). New lactone and xanthone derivatives produced by a mangrove endophytic fungus *Phoma* sp. SK3RW1M from the South China Sea. Helv. Chim. Acta.

[20] Shen Y., Xu Q.L., Cheng P., Liu C.L., Lu Z.Y., Li W., Wang T.T., Lu Y.H., Tan R.X., Ge H.M. (2017). Aromatic polyketides from a caterpillar associated *Alternaria* sp.. Tetrahedron Lett..

[21] Sheldrick G.M. (1996). SADABS, Software for Empirical Absorption Correction.

[22] Sheldrick G.M. (1997). SHELXTL, Structure Determination Software Programs.

[23] Sheldrick G.M. (1997). SHELXL-97 and SHELXS-97, Program for X-ray Crystal Structure Solution and Refinement.

